# Lasers treatment for traumatic facial tattoo

**DOI:** 10.1007/s10103-022-03658-1

**Published:** 2022-12-29

**Authors:** Qing Yang, Ping Xue, Xing Fan, Yue Yin, Hui Dang, Wei Li, Baoqiang Song

**Affiliations:** Department of Plastic and Reconstructive Surgery, The First Affiliated Hospital of Air Force Medical University (Xijing Hospital), Xi’an, 710032 China

**Keywords:** Q-1064 nm laser, CO_2_ ablative fractional laser, Traumatic facial tattoo, Scar

## Abstract

Effective treatment for traumatic tattoo is lacking. We aimed to compare the effectiveness and safety between Q-1064 nm laser as monotherapy and alternating Q-1064 nm laser with CO_2_ AFL in treating traumatic facial tattoo (black or blue color) and accompanied scars. Clinical data of 98 patients were grouped and analyzed based on the different treatment lasers. Tattoo clearance was evaluated with a 4-point scale, and scar improvement was analyzed with modified VOSAS scores. Patient satisfaction of the tattoo clearance and scar improvement, and treatment-related complications were analyzed. Significant increased clinical effects with patient satisfaction and decreased worsened scar were confirmed with the two alternated lasers, relative to those with Q-1064 nm laser alone (*P* < 0.05). Consequently, alternating Q-1064 nm laser and CO_2_ AFL treatment could be used for traumatic facial tattoo with black or blue color removal safely and effectively.

## Introduction

Traumatic Facial tattoos may negatively alter a person’s appearance due not only to the acquired mostly black or blue pigmentation induced by the depositing of exogenous pigmented particles (e.g., metal, dust, carbon particles, or gunpowder) but also to the scarring caused by explosion or traumatic events [1, 2].

Nanosecond Q-1064 nm is often used as the first-line treatment for black or blue tattoo removal by selective photothermol and photomechanical disruption of the target tattoo pigment particles [3–5], and the risk of the consequential scarring is about 0.28 to 8.65% [6]. In addition, the risk of scarring is prone to increase when the foreign particles are carbon or gunpowder [7]. So, both tattoo removal and scarring improvement should be considered simultaneously for traumatic scarred tattoo treatment.

CO_2_ ablative fractional laser (AFL) is the first-line treatment for scarring improvement with safety and efficacy [8, 9]. These characteristics may indicate that CO_2_ lasers can be used as a supplemented method to improve the scar when Q-1064 nm laser is used for tattoo removal. However, there are few studies regarding scarring characteristic assessment before and after laser tattoo removal.

In the present retrospective cohort study, we aimed to compare the clinical efficacy and safety between Q-1064 nm laser monotherapy with alternating Q-1064 nm laser and CO_2_ AFL treatments for traumatic facial tattoos.

## Materials and methods

We performed this retrospective study in which we included patients diagnosed with traumatic facial tattoos, underwent four laser treatment sessions, and had whole medical records from January 2012 to June 2021 at the Plastic Surgery Department of the First Affiliated Hospital of Air Force Military Medical University.

Patients were included in group A who received Q-1064 nm laser (MedLite C6, HOYA ConBio, Fremont, CA, USA) as mono-therapy with the following settings: spot size, 3–4 mm; fluence, 4–6 J/cm2; frequency, 10 Hz [2]. The treatment endpoint was white frost on the local skin surface.

Patients were included in group B who accepted alternating treatments of Q-1064 nm laser (1st and 3rd) as in group A and CO_2_ AFL (2nd and 4th) (Ultrapulse, Lumenis, Yokneam, Israel). The pulse energy of the CO_2_ AFL in Deep FX mode (spot size, 120 μm) ranged from 17.5 to 30.0 mJ/pulse, 5% density, and 150-Hz frequency [8, 10]. The immediate endpoint of the CO_2_ AFL treatment was slight swelling and erythema. After treatment, an ice pack was applied to relieve any discomfort. All the patients received a sterile collagen mask (Chuangfukang, Guangzhou Chuanger Biotechnology Co., Ltd, China) once daily for 1 week to promote wound healing. During the treatment period, all the patients were asked to use sunscreen to avoid sun exposure.

Two observer questionnaires (clinical tattoo clearance efficacy, observer scar assessment scale (OSAS) score) and side effects were completed by two independent physicians from patient medical records. One questionnaire (patient satisfaction with tattoo clearance and scar improvement) was performed by patients or their guardians in May 2021.

This study focused on three topics: clinical efficacy, patient satisfaction, and side effects.

### Clinical efficacy evaluation

#### Clinical tattoo clearance efficacy and observer scar assessment scale (OSAS) score

Two independent physicians assessed the clinical tattoo clearance efficacy by comparing the final and pretreatment photographs. based on a 4-point scale:(1, 0–25%, poor; 2, 26–50%, fair; 3, 51–75%, good; 4, 76–100%, excellent) [4]. The clinical effectiveness rate of tattoo clearance was calculated using the formula: [(number of patients with “good improvement” + number of patients with “excellent improvement”)/total number of patients] × 100%.

OSAS score was used to analyze skin and scar characteristics of the tattoo area including pigmentation, vascularity, thickness, relief, pliability, and surface area before and after final treatment based on medical file records. We excluded pigmentation scoring to reduce tattoo color interference as the exogenous particles in the patient treatment area appeared black or blue. Each was scored on a scale from 1 (normal) to 10 (worst imaginable), so that the total modified OSAS score ranged from 5 (normal skin appearance) to 50 (worst scar appearance) points. [11].

#### Patient satisfaction with tattoo clearance and scar improvement

Patients satisfaction with clearance of tattoo and improvement of scar scales for self evaluation on a 0–10 assessment scale (0, no change; 10, excellent) was collected by telephone survey and analyzed [8].

### Evaluation of side effects

The number of adverse events such as blisters, local infection, hypo- or hyperpigmentation, scar worsening, skin allergies, and other adverse events was collected and analyzed in all the 98 patients based on medical files after the four treatment sessions.

### Statistical analysis

The data were recorded in the form of mean $\pm$ SD. Unpaired samples *t*-test was used to evaluate the differences between the two groups in tattoo clearance, scar improvement, and patient satisfaction at the last visit. Fisher’s exact test and chi-squared test compared the two groups for adverse events at the final visit. A difference with *P* < 0.05 was considered statistically significant. All the analyses were performed with SPSS software version 22.0.

## Results

### Patient demographics

A total of 98 patients (39 males and 59 female) aged 18 to 45 years old and who received laser treatment were included in this study under the selection criteria.

The 52 patients in group A were treated with Q-1064 nm laser alone, and the 46 patients in group B were treated using alternating treatments of Q-1064 nm laser (1st and 3rd) and CO2 AFL (2nd and 4th). All the patients had Fitzpatrick skin type III or IV. Patient information is shown in Table 1.

**Table 1:** Demographics and tattoo characteristics of the patients

	Group A (*n* = 52)	Group B (*n* = 46)	*P*-value
Age (mean ± SD) (years)	25 ± 10	22 ± 9	> 0.05
Sex (*n*)			> 0.05
Male	21	18	
Female	31	28	
Fitzpatrick skin type (*n*)			> 0.05
III	25	20	
IV	27	26	
Age of tattoo, mean ± SD (years)	3 ± 9	3 ± 4	> 0.05
Size (cm^2^)			> 0.05
6–25	4	3	
26–50	12	9	
> 50	36	34	
Causes of injury (*n*)			> 0.05
Contusion	41	23	
Blast	11	13	

*SD*, standard deviation

### Analysis of clinical efficacy of tattoo clearance and scar improvement

The number of patients in group A with excellent, good, fair, and poor tattoo clearance was 4 (7.69%), 19 (36.54%), 25 (48.08%), and 4 (7.69%) cases, whereas that in group B was 8 (17.39%), 26 (56.52%), 12 (26.09%), and 0 (0%) cases, respectively. The clinical tattoo clearance effectiveness rates for the Q-1064 nm laser alone and alternating Q-1064 nm laser with CO_2_ AFL were 44.23 and 73.91%, respectively, with a significant difference between the two groups (*P* < 0.001) (Table 2).

**Table 2:** Evaluation of tattoo removal, patient satisfaction, and adverse events between groups

			Group A (*n* = 52)	Group B (*n* = 46)	*P*-value
Clinical effectiveness rates of tattoo removal (%)			44.23	73.91	< 0.001
Patient satisfaction score (mean ± SD)					
	Tattoo clearance score		4.51 ± 0.67	6.85 ± 0.39	< 0.001
	Scar improvement score		3.36 ± 0.67	6.02 ± 0.17	< 0.001
Adverse events *n*(*%*)					
	Blister		7 (13.46)	1 (2.17)	> 0.05
	Hypopigmentation		4 (7.69)	1 (2.17)	> 0.05
	Hyperpigmentation		6 (11.54)	1 (2.17)	> 0.05
	Worsened scar		10 (19.23)	2 (4.35)	< 0.05

*SD*, standard deviation

The OSAS scores related to scar characteristics in the tattoo region were compared, and no significant differences were observed between the two groups before treatment (*P* > 0.05).

**Fig. 1:**
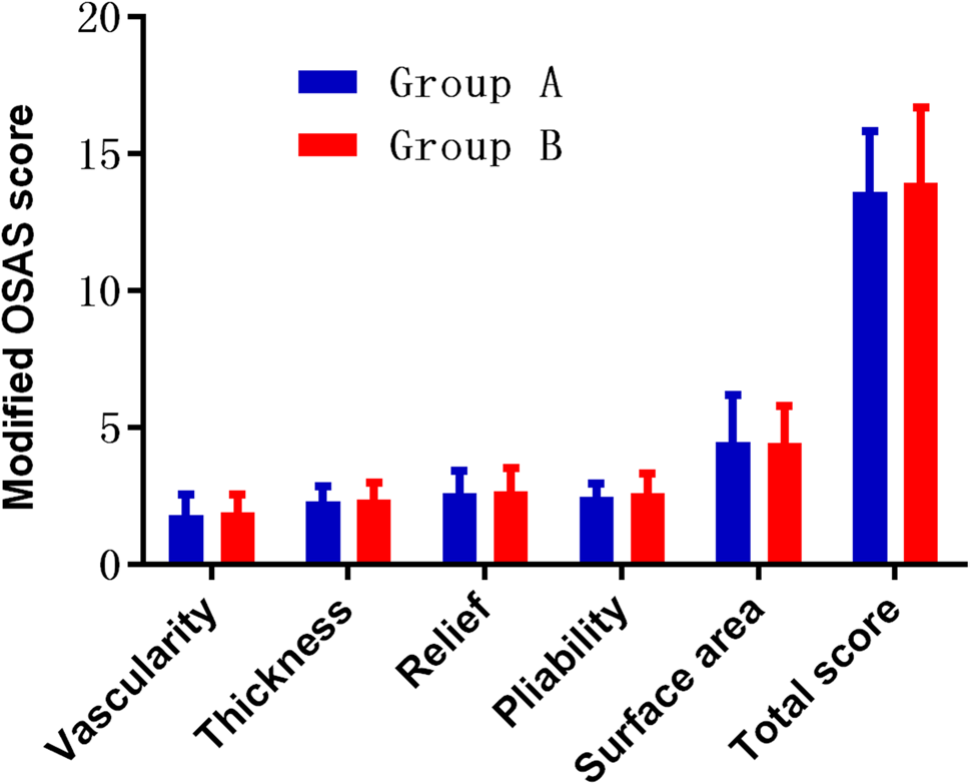
Comparison of modified OSAS scores before treatment between groups. OSAS, observer scar assessment scale

**Fig. 2:**
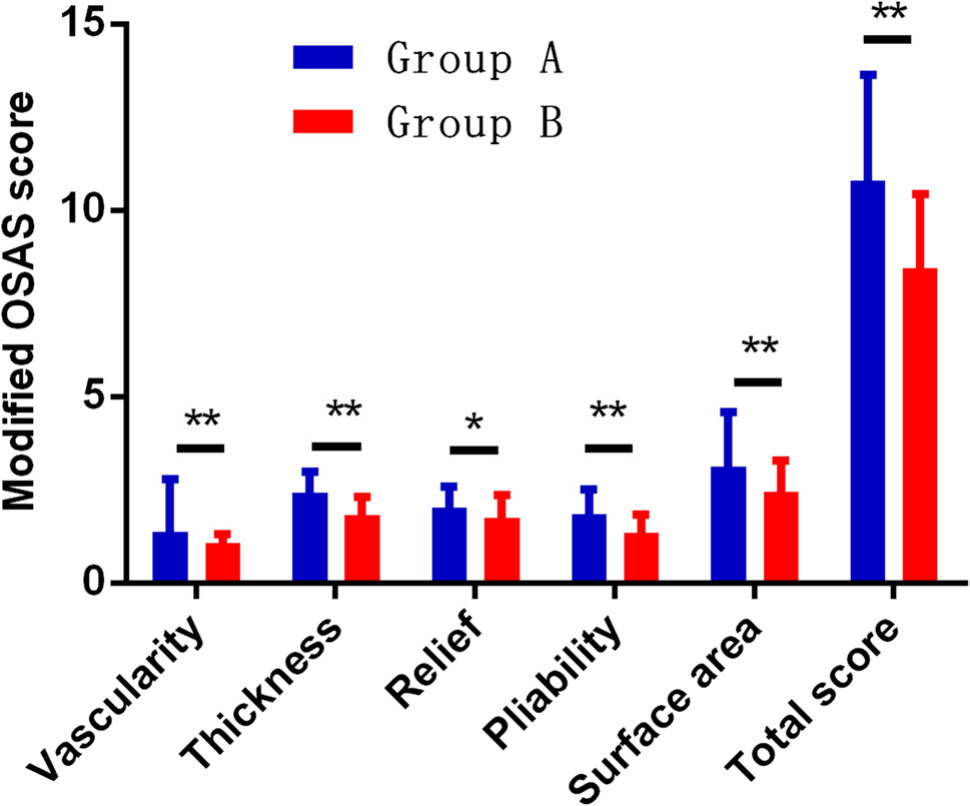
Comparison of modified OSAS scores after treatment between groups. OSAS, observer scar assessment scale. *P < 0.05 and ** *P* < 0.01

The analyses of OSAS scores obtained at final visit indicated that the value of significantly decreased the values of vascularity, relief, pliability, and surface area and total OSAS score in both laser treatment groups and scar thickness in group B than before (*P* < 0.01). The value of scar thickness in group A after treatment (2.42 ± 0.57) was insignificantly higher than before treatment (2.30 ± 0.54) (*P* > 0.05). Moreover, alternating Q-1064 nm laser with CO_2_ AFL treatment significantly decreased the values of vascularity, thickness, relief, pliability, surface area, and total OSAS than Q-1064 nm laser monotherapy (*P* < 0.05) (Figs. 1 and 2).

### Patient satisfaction with tattoo clearance and scar improvement

The satisfaction scores were 4.51 ± 0.67 (group A) versus 6.85 ± 0.39 (group B) for tattoo clearance, and 3.36 ± 0.67 (group A) versus 6.02 ± 0.17 (group B) for scar improvement, which were significantly different between the two groups (*P* < 0.001) (Table 2).

### Adverse events

After four treatment sessions, the incidence rates of blister, hypopigmentation, and hyperpigmentation in group A were not significantly different from that in group B (*P* > 0.05). Blisters in all the cases faded in 2 weeks. But four cases in group A and one in group B after blister generated transient hyperpigmentation, which disappeared 6 months after the final treatment. Permanent hypopigmentation was observed 6 months after the final treatment.

**Fig. 3:**
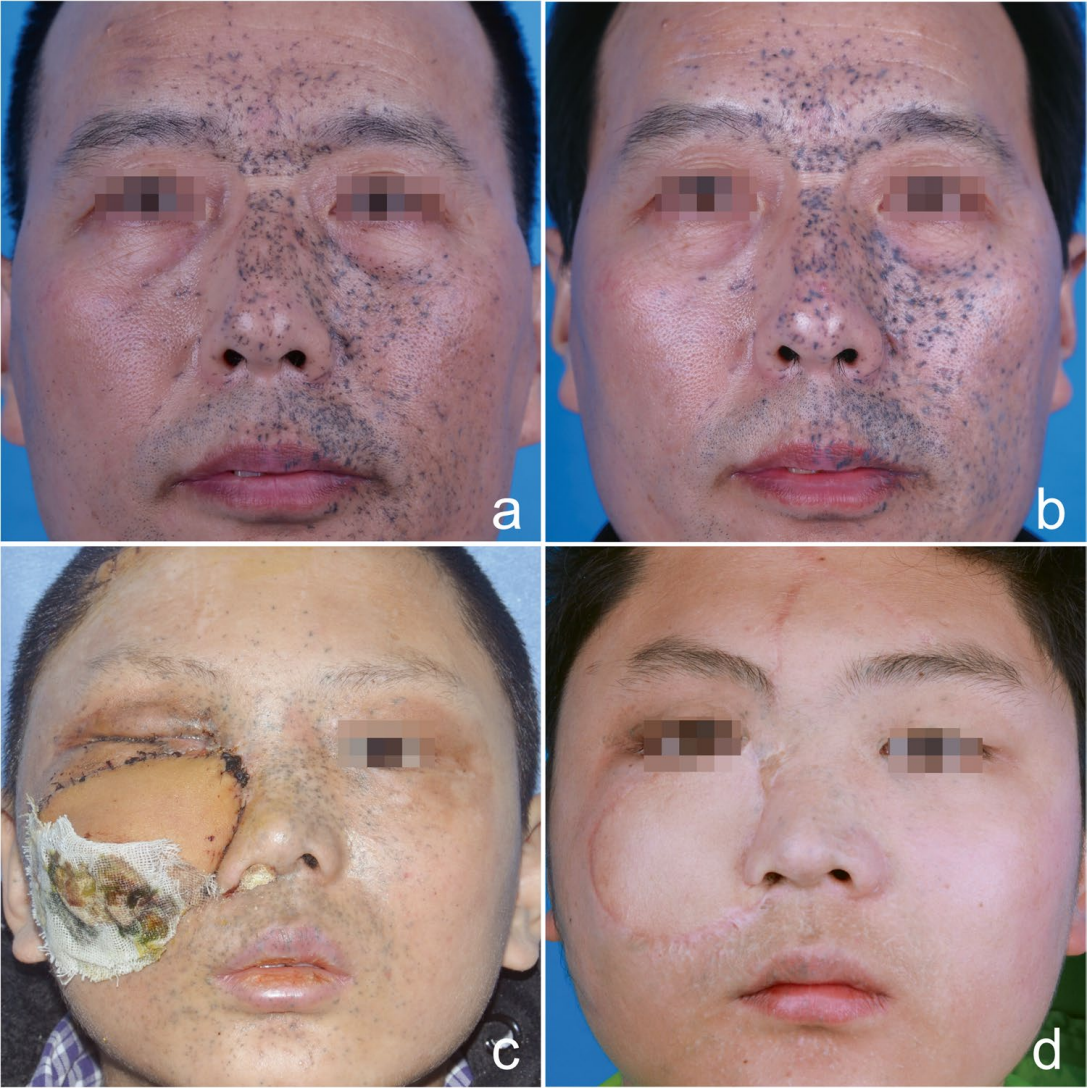
Traumatic tattoo due to blast. **a** Baseline facial profile in group A and **b** poor tattoo clearance achieved, punctate atrophic scar was unchanged after final treatment. **c** Baseline facial profile in group B and **d** excellent tattoo clearance achieved. The apparent punctate hypertrophic scar and hypo-pigmentation formed by skin splash and bulla after Q-1064 nm (1st) treatment. The scar was smoothed after two sessions of Co2 AFL treatment, but the hypo-pigmentation was almost unchanged

**Fig. 4:**
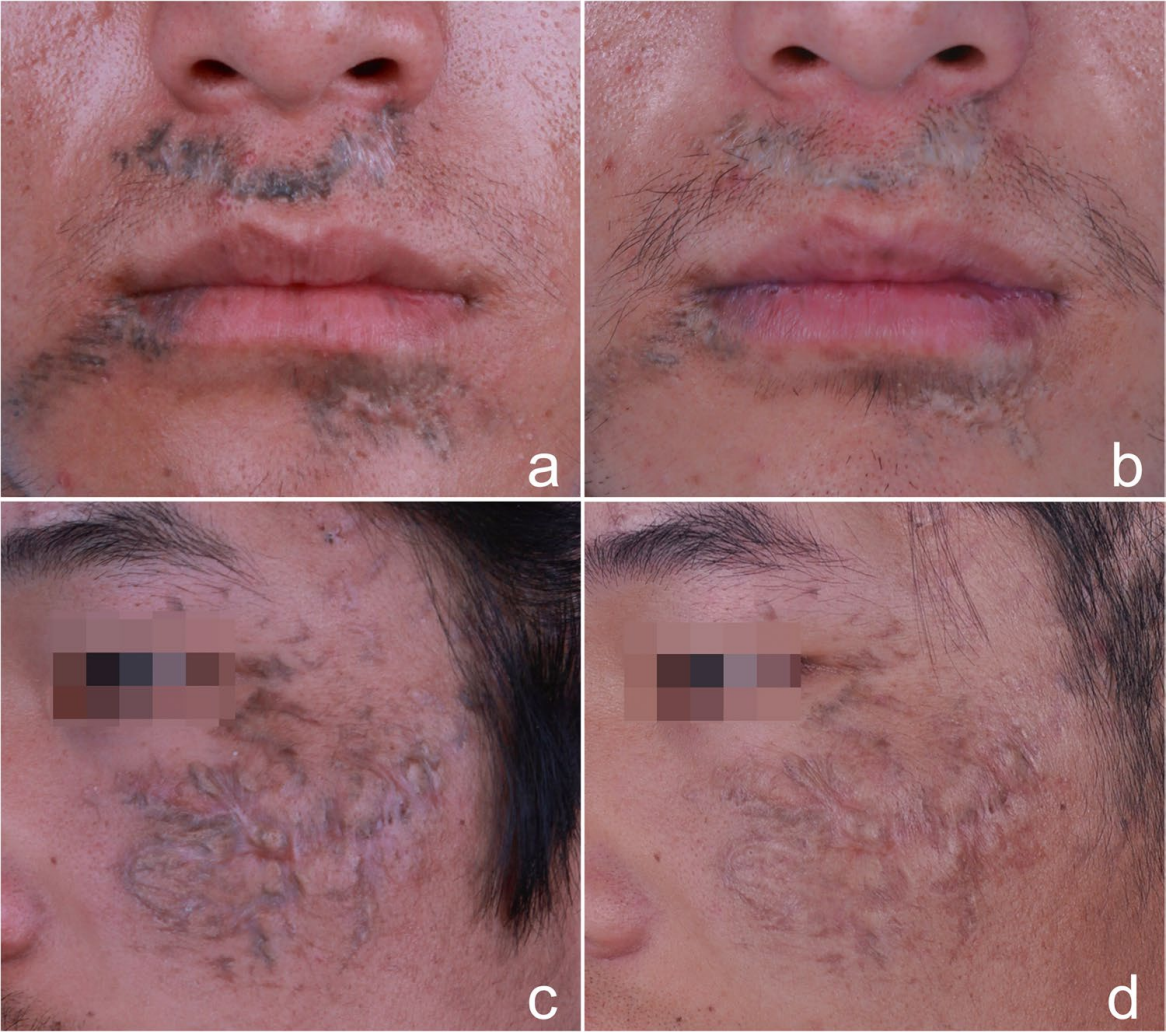
Traumatic tattoo due to contusion. **a** Baseline facial profile in group A and **b** good tattoo clearance achieved, mostly atrophic scar was unchanged, but with hypo-pigmentation after final treatment. **c** Baseline facial profile in group B and **d** excellent tattoo clearance and improvement in scar observed after final treatment

The worsened scar of 19.23% (10/52, including seven cases of hypertrophic scar and three cases of punctate depressed scar) in group A was significantly higher than that of 4.35% (2/46, including one case of hypertrophic scar and one case of punctate depressed scar) in group B (*P* < 0.05) (Table 2, Figs. 3 and 4) after the final treatment.

## Discussion

This study shows a significant increase in tattoo clearance rate and improvement in accompanied scar and a decrease in consequent complication when CO_2_ AFL is added to nanosecond Q-1064 nm laser as an alternating treatment for traumatic tattoo removal.

For CO_2_ AFL with epidermal pigmentation ablation followed by Nd:YAG laser for concentrated tattoo disruption at the same session, a higher cosmetic black tattoo clearance was confirmed, but a higher risk of consequential scar was observed. [12]. Even though the lower energy of Ultrapulse CO_2_ AFL setting was lowered to 3 J/cm^2^ and 5% coverage, the incidence of scar was as high as 20% [13]. However, there was a lack of study on traumatic tattoo removal. The risk of scar after combined treatment is theoretically higher than Q-1064 nm laser alone.

Because, the traumatic foreign particles (especial for carbon particles and gunpowder) blasting under the high-energy laser might prone to produce tissue splatter and consequential new scars due to the secondary relatively severe microexplosion under the skin [14, 15]^.^ So, in our study, we did use alternating laser treatment instead of combined treatment for traumatic facial black tattoo to minimize tissue thermal damage and lower the risk of complications.

In this present study, consist with clinical efficacy of tattoo clearance, the patient satisfaction about tattoo removal (4.51 ± 0.67) after Q-1064 nm laser treatment was lower than that (6.85 ± 0.39) in group. This may be due to the characteristic of Q-1064 nm laser. Q-1064 nm laser could penetrate nearly about 4 mm for skin tissues and disrupt blue or black pigmented tattoo with particle diameters of 10–40 μm^3^ from its photomechanical and photothermal effects [16]. But lager or deeper particles in the skin are difficult to breakdown, which required more sessions to fine [17]. CO_2_ AFL laser was set at 15 to 30 mJ/pulse, the penetration depth was approximately 1–3 mm, and the micro-ablation size was around 120 μm for human skin [8]. Therefore, deeper and lager sized, around 100 μm particles would be vaporized and extruded. Besides tattoo removal, CO_2_ AFL laser is associated with decreased consequential scar formation and increases patient satisfaction due to the collagen remodeling to smooth scar texture. Consistent with significant lowering of the worsened scar with the alternated lasers (CO_2_ AFL and Q-1064 nm) relative to the Q-1064 nm laser alone, the vascularity, thickness, relief, pliability, surface area, and the total revised OSAS score that were representative of scar characteristics were also significantly decreased with the alternated lasers, primarily because of the remodeling of scar collagen (*P* < 0.05). The percentiles of blister, hypo-, and hyperpigmentation did not significantly differ between the two treatment groups (*P* > 0.05).

The Co_2_ AFL alone for traumatic tattoo has not been recommended as a prior treatment except for recalcitrant and allergic tattoo compared with Q-1064 nm alone [18]. which may be due to the limited safety fractional ablation settings.

Over-vaporized skin by Co_2_ AFL would lead to permanent scar. So, it is a dilemma for physicians to balance the increased ablated pigment-loaded tissue and the higher risk of consequential complication.

Therefore, find the appropriate laser energy parameter for Co_2_ AFL as a alternating and supplemental method with Q-1064 nm to remove these foreign particles utmostly and safely by graded energy study will be the further research.

The limitation of our study is the lack of a limited number of enrolled patients, longer follow-up period, and lack of histological examination for the two different laser methods to compare the safety and clinical effectiveness of the above two laser treatment approaches for traumatic tattoos. However, this study provided an alternating laser treatment procedure for black and blue traumatic tattoo removal with safety and efficacy and with a relative objective and subjective evaluation.

## Conclusions

In this study, patients with traumatic facial tattoo improved with 73.91% clinical efficacy of tattoo clearance, and total OSAS scores decreased from 13.93 ± 2.75 to 8.46 ± 1.99, and with 4.35% risk of worsened scar after four alternated laser treatment sessions. So, alternated laser treatment with CO_2_ AFL and Q-1064 nm laser could provide an option for better tattoo clearance and scar improvement with lower risk of complication.
